# Nanomaterials: certain aspects of application, risk assessment and risk communication

**DOI:** 10.1007/s00204-017-2144-1

**Published:** 2017-12-22

**Authors:** Peter Laux, Jutta Tentschert, Christian Riebeling, Albert Braeuning, Otto Creutzenberg, Astrid Epp, Valérie Fessard, Karl-Heinz Haas, Andrea Haase, Kerstin Hund-Rinke, Norbert Jakubowski, Peter Kearns, Alfonso Lampen, Hubert Rauscher, Reinhilde Schoonjans, Angela Störmer, Axel Thielmann, Uwe Mühle, Andreas Luch

**Affiliations:** 1 Department of Chemical and Product Safety, German Federal Institute for Risk Assessment (BfR), Max-Dohrn-Strasse 8-10 10589 Berlin, Germany; 2 Department of Food Safety, German Federal Institute for Risk Assessment (BfR), Max-Dohrn-Strasse 8-10 10589 Berlin, Germany; 3 Department of Inhalation Toxicology, Fraunhofer-Institute for Toxicology and Experimental Medicine (ITEM), Nikolai Fuchs Strasse 1 30625 Hannover, Germany; 4 Department of Risk Communication, German Federal Institute for Risk Assessment (BfR), Max-Dohrn-Strasse 8-10 10589 Berlin, Germany; 5 Laboratoire de Fougères, French Agency for Food, Environmental and Occupational Health and Safety (ANSES), 10B Rue Claude Bourgelat 35306 Fougères Cedex, France; 6 Fraunhofer Institute for Silicate Research ISC, Neunerplatz 2 97082 Würzburg, Germany; 7 Fraunhofer Institute for Molecular Biology and Applied Ecology IME, Auf Dem Aberg 1 57392 Schmallenberg, Germany; 8 Division 1.1 Inorganic Trace Analysis, Federal Institute for Materials Research and Testing (BAM), Richard-Willstaetter-Str. 11 12489 Berlin, Germany; 9 OECD Environment, Health and Safety Division 2, rue Andre-Pascal 75775 Paris Cedex 16, France; 10 Joint Research Centre (JRC) of the European Commission, Directorate Health, Consumers and Reference Materials, Via E. Fermi, 2749 21027 Ispra, Italy; 11 Scientific Committee and Emerging Risks Unit, European Food Safety Authority (EFSA), Via Carlo Magno 1a 43126 Parma, Italy; 12 Fraunhofer Institute for Process Engineering and Packaging IVV, Giggenhauser Strasse 35 85354 Freising, Germany; 13 Fraunhofer Institute for Systems and Innovation Research ISI, Breslauer Strasse 48 76139 Karlsruhe, Germany; 14 Fraunhofer Institute for Ceramic Technologies and Systems IKTS, Winterbergstr. 28 01277 Dresden, Germany

**Keywords:** Nanomaterials, Toxicity, Ecotoxicity, Standardization, Exposure

## Abstract

Development and market introduction of new nanomaterials trigger the need for an adequate risk assessment of such products alongside suitable risk communication measures. Current application of classical and new nanomaterials is analyzed in context of regulatory requirements and standardization for chemicals, food and consumer products. The challenges of nanomaterial characterization as the main bottleneck of risk assessment and regulation are presented. In some areas, e.g., quantification of nanomaterials within complex matrices, the establishment and adaptation of analytical techniques such as laser ablation inductively coupled plasma mass spectrometry and others are potentially suited to meet the requirements. As an example, we here provide an approach for the reliable characterization of human exposure to nanomaterials resulting from food packaging. Furthermore, results of nanomaterial toxicity and ecotoxicity testing are discussed, with concluding key criteria such as solubility and fiber rigidity as important parameters to be considered in material development and regulation. Although an analysis of the public opinion has revealed a distinguished rating depending on the particular field of application, a rather positive perception of nanotechnology could be ascertained for the German public in general. An improvement of material characterization in both toxicological testing as well as end-product control was concluded as being the main obstacle to ensure not only safe use of materials, but also wide acceptance of this and any novel technology in the general public.

## Introduction

The increased application of nanomaterials (NMs) in production (Bekker et al. 2013; Kreider et al. 2015) and construction (Hanus and Harris 2013; Hincapie et al. 2015), as well as in a wide range of nano-enabled consumer and medical products (Vance et al. 2015) has resulted in an enhanced exposure of humans and the environment. Besides the ingestion of NMs with food (Szakal et al. 2014), direct dermal contact (Gulson et al. 2015) and inhalation (Donaldson and Seaton 2012) represent scenarios that humans may encounter. The latter is currently considered the most relevant. Environmental exposure derives mostly from material aging and waste (Mitrano et al. 2015; Neale et al. 2013). Despite the widespread sources of NM release (Lankone et al. 2017; Wagener et al. 2016), it is subject to debate whether there is relevant human exposure to NMs in daily life (Ding et al. 2017; Goehler and Stintz 2014). With respect to toxicity, a multitude of studies have failed to reveal a risk of materials in the nano-dimension per se (Gebel et al. 2013; Krug 2014). Generic mechanisms such as dust overloading of the lungs (Laux et al. 2017a) and frustrated phagocytosis (Murphy et al. 2012) are considered relevant for NMs, too. Regulatory and standardization measures for nano-scale materials have not been implemented consistently (Garduno-Balderas et al. 2015). Nevertheless, the question remains to what extent and how existing test guidelines should be adapted for NMs in order to differentiate their effects to those of bulk materials. Several novel analytical techniques have been developed recently and those may alleviate the current lack in NM characterization (Antonio et al. 2015; Buchner et al. 2014). A missing size estimation of NMs often prevents the comparison of their toxicity to bulk material (Krug 2014). The ongoing scientific discussion has resulted in uncertainties for the general public regarding a potential risk of NMs (Gupta et al. 2013), in particular when used in food products (Hallman and Nucci 2015). Different stakeholders, representing the areas material research, regulation, toxicology, ecotoxicology and risk communication, have reviewed these topics in the aftermath of a workshop organized by the German Federal Institute for Risk Assessment (BfR) together with the Fraunhofer-Alliances Food Chain Management and Nanotechnology.

## Industrial use and standardization of nanomaterials

**Fig. 1:**
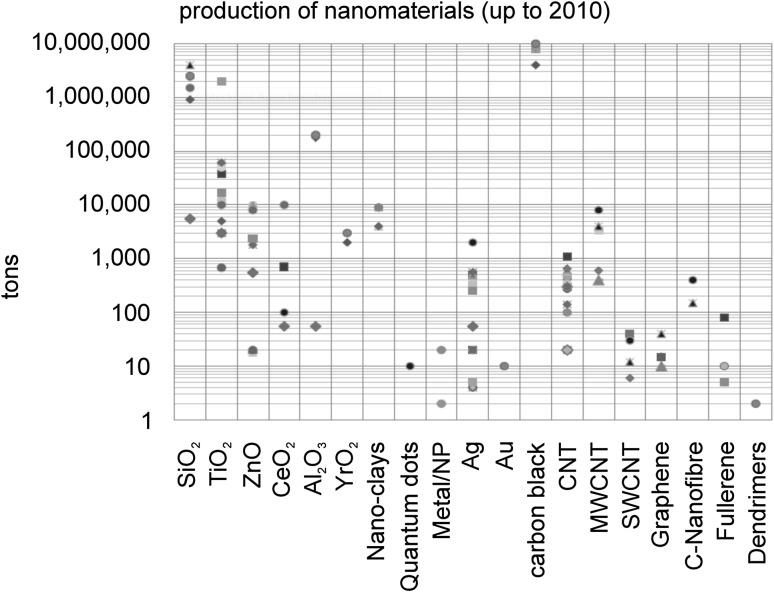
Production of nanomaterials up to 2010. Different symbols represent estimations by different sources reviewed in Haas (2013)

**Fig. 2:**
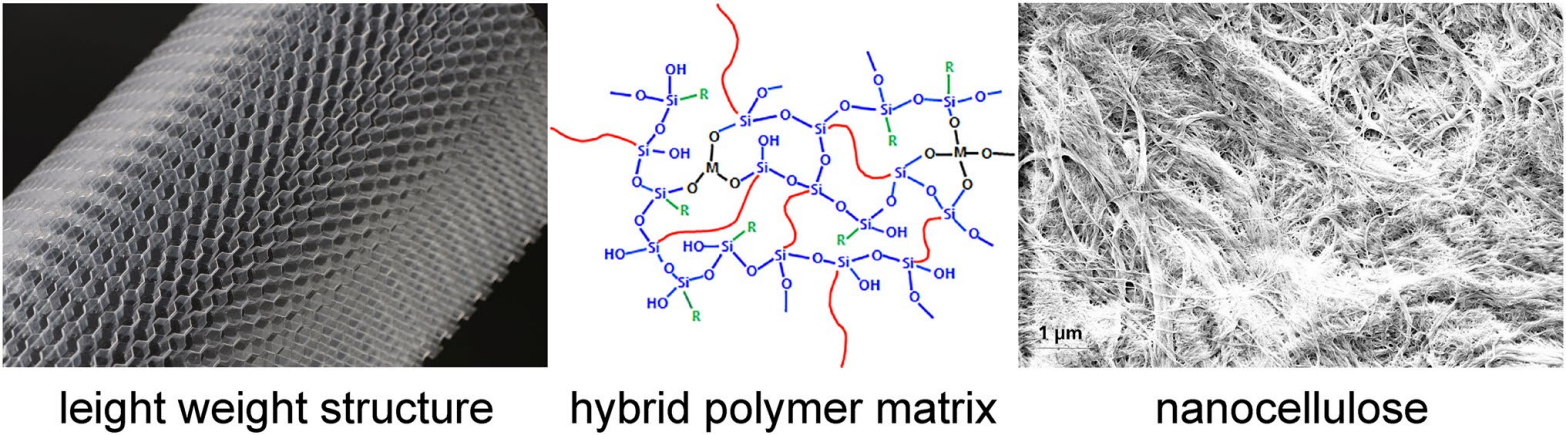
Example for the application of nanocellulose as a filler in coatings for light-weight structures

Due to their unique size-dependent properties, NMs exert a ground breaking impact on diverse application areas ranging from construction industry via daily life products to applications in medicine and healthcare. The current world market of an estimated 150–200 billion euro is mainly represented by classical NMs such as carbon black, silicon dioxide (SiO_2_), titanium dioxide (TiO_2_) and silver (Ag) that account for more than 90% of the production volume, while the use of new NMs such as fullerenes, carbon nanotubes and dendrimers still remains at a low level (Fig. 1) (Haas 2013). Among the new players nanocellulose (Cowie et al. 2014) and graphene (Cheng et al. 2017; Ricci et al. 2017) are the most promising candidates, the former being produced by generating individual cellulosic fibers from renewable sources such as wood or algae. Further transformation of fibers leads to micro- and nanofibrils (Rebouillat and Pla 2013) that may be used, for example, as a light-weight filler (Fig. 2), for packaging coatings, as a replacement of plastic packaging, and in cement. Applications of graphene range from the increasing replacement of indium tin oxide for energy conversion in touch screen applications (Janas and Koziol 2014), the preparation of high-strength epoxy/graphene nanocomposites for automotive applications (Wei et al. 2015) to functional inks for flexible electronics embedded into textiles or other everyday commodities (Capasso et al. 2015). Established methods for graphene production comprise unzipping of carbon nanotubes (Kosynkin et al. 2009) and molecular assembly by carbon molecular beam epitaxy (Park et al. 2010). Similar to graphene, most NMs can be manufactured either top down by disintegration of larger units or bottom up by assembly of molecular entities. SiO_2_, the NM with the highest production volume (Haas 2013), is usually produced purpose-specific, either by pyrogenic flame synthesis, e.g., for the use as thickener, or by precipitation, in case of its final use in paints, coatings or papers. The versatile applications of TiO_2_ nanoparticles include photocatalysis in transparent hybrid polymer coatings (Lee et al. 2010), besides its long known use as UV filter in cosmetic products and textiles. More recent developments include protective acrylic coatings of buildings, to which TiO_2_ is added following a modification with Al_2_O_3_ and second polyhedral oligomeric silsesquioxanes for a better dispersion in water-based formulations (Godnjavec et al. 2012).

The definition of an NM is based on different criteria, according to its foreseen purpose in industry, research, or regulation. According to the International Organization for Standardization (ISO) “nanotechnology” is the “application of scientific knowledge to manipulate and control matter predominantly in the nanoscale (i.e., approximately from 1 to 100 nm) to make use of size- and structure-dependent properties and phenomena distinct from those associated with individual atoms or molecules, or extrapolation from larger sizes of the same material”. ISO also classifies and categorizes NMs systematically. It defines an NM as material with any external or internal structures or surface structures in the nanoscale (ISO 2015). Furthermore, the European Commission (EC) has recommended a definition of the term “nanomaterial” specifically to be used for regulatory purposes (EC 2011). According to this recommendation, a material, regardless of its origin (i.e., natural, incidental or manufactured), is considered being an NM if it contains 50% or more of unbound, agglomerated or aggregated particles with one or more external dimensions in the size range between 1 and 100 nm. As an exemption from this rule, for fullerenes, graphene flakes and single-wall carbon nanotubes the lower size limit remains ineffectual, while the upper limit of 100 nm still applies. Furthermore, the 50% threshold referring to the number of particles with a size between 1 and 100 nm may be lowered down to 1% in specific cases and whenever warranted due to concerns triggered by environmental, health, and safety concerns or by competitiveness. Once adopted for regulatory purposes in order to establish legal clarity, the EC recommendation requires appropriate analytical measures to determine number-based particle size distributions at the nanoscale and well beyond 100 nm. Currently, this requirement still represents a considerable challenge. Even more so, as NMs suffer from the tendency to agglomerate in the atmosphere or in liquid media, a feature that often hinders their identification by particle analysis techniques. Agglomeration may also lead to the loss of nanoscale-mediated properties such as increased surface area (and reactivity), translucency and specific particle motion behavior while being in dispersion.

## Regulation of nanomaterials in the areas of chemicals, biocides, consumer products and food

Within the European Union, all chemicals and their use in products for which no other specific regulation exists are subject to the Regulation EC No 1907/2006 concerning the Registration, Evaluation, Authorization and Restriction of Chemicals (REACH) (EC 2006). This includes high-volume substances such as SiO_2_ or TiO_2_, both of which are mostly used in the manufacturing of, e.g., coatings or composites. NMs are not explicitly mentioned in the REACH framework, but since it applies to chemical substances in any form and configuration, NMs are being covered as well. The European Chemicals Agency (ECHA) has published guidance documents on the information requirements and safety assessments according to REACH. This also comprises specific requirements for substances that fall into the recommendation of the EC for the definition of NMs, such as data on physicochemical properties, toxicology and toxicokinetics as well as appropriate safety assessments. A nanospecific occupational exposure evaluation is also included and recommendations on protective personnel equipment are given for the case that residual exposure cannot be avoided by application of other means. As suitable dose metrics for inhalation exposure to NM, mg/cm^2^, cm^2^/m^3^ and particle number/cm^3^ are proposed, the latter being of particular relevance also for fibers (ECHA 2016). A fiber is considered hazardous when thinner than 3 µm and longer than ~ 20 µm and no biodegradation in the lungs by dissolving or breaking is possible (Donaldson and Tran 2004). For materials such as carbon nanotubes, silicon carbide and fluoro-edenite retention in the parietal pleura has been associated with the development of cancer following inhalation (Donaldson et al. 2010; Grosse et al. 2014).

The use of an NM as active or non-active ingredient of a biocidal product must also be authorized based on a separate, nanospecific risk assessment and requires specifying the intended application areas such as the antimicrobial furnishing of products (EC 2012). In case of the bactericidal nanosilver (nano-Ag), hints on its accumulation in humans as well as on the development of bacterial cross-resistances towards antimicrobials that are applied in the treatment of patients have led to the recommendation to refrain from its application in consumer products (BfR 2009; Schäfer et al. 2013).

Cosmetic products are an example of a specific regulation of consumer products. The European regulation for cosmetic products requires a notification of the EC along with information on the toxicological profile of the NM as well as relevant safety data (EC 2009). Of note is that the legislation has a somewhat different definition of NM to that of the EC recommendation, explicitly mentioning manufactured materials and forgoing the definition of a fraction in the nanoscale. The use of NMs as UV filters, colorants and preservatives must be explicitly approved based upon specific safety assessments. Here, the Notes of Guidance for the testing of cosmetic substances and their safety evaluation, as issued by the Scientific Committee on Consumer Safety, are applicable (SCCS 2012). In addition, specific characteristics of NMs need to be considered as well (SCHER 2009). For the dermal application of nano-TiO_2_ in concentrations of up to 25%, used as UV-filter in sunscreens on healthy or sunburnt skin, the risk assessment revealed the lack of dermal absorption and thus no adversity in humans (SCCS 2014). However, this does not apply to similar products that might be inhaled, such as powders or spays. The risk assessment for NMs is still to be developed further and requires in particular the consideration of the toxicokinetic behavior of respirable particles. The EC has authorized the use of nano-TiO_2_ as UV-filter in cosmetic products under specific conditions only, and in adherence to an appropriate and nanospecific risk assessment. Notably, a possible risk is attributed to cosmetic spray products generating respirable aerosols (EC 2016).

For NMs in agriculture, food and feed, authorization is granted by the EC and member states of the European Union. The risk assessment is based on the sectorial guidance documents of the European Food Safety Agency (EFSA), and the corresponding guidance document for nanotechnology and nanoscience in the food and feed chain (EFSA 2011). The uses covered in such evaluations comprise, for instance, applications as ingredients in animal feed, food, food additives and for food packaging materials. According to the EFSA guidance, the characteristics of an engineered nanomaterial (ENM) should ideally be determined at five different stages: (1) as manufactured material (pristine state); (2) upon delivery in food/feed products; (3) as being present in the food or feed matrix; (4) as being present in the medium for toxicity testing, and (5) as being present in fluids of the human or animal body. Six case scenarios were established for exposure assessment based on particle persistence and ingestion (Table 1) (EFSA 2011). The EFSA guidance is currently being updated.

**Table 1:** Case scenarios for the risk assessment of nanomaterials in agriculture, feed and food (EFSA 2011)

Case	Scenario description
1. No persistence of engineered nanomaterials in preparations/formulations as marketed	For nanotechnology applications where convincing evidence is provided, demonstrating, by appropriate analytical methods that the ENM is completely degraded/solubilized to non-nanoform, the EFSA Guidance for non-nanoforms for the specific intended use should apply, and this ENM Guidance would no longer apply
2. No migration from food contact materials (i.e., no exposure)	Where evidence is provided convincingly demonstrating, by appropriate analytical methods that there is no migration, the risk assessment could be based on the information that there is no exposure to the ENM via food and therefore there is no toxicological concern
3. Complete transformation of engineered nanomaterials into a non-nanoform in the food/feed matrix before ingestion	When evidence is provided convincingly demonstrating, by appropriate analytical methods, that transformation of the ENM into a non-nanoform in the food/feed matrix is judged to be complete (i.e., non-nanoform degradation products are present) before ingestion, then EFSA Guidance for non-nanoforms for the specific intended use should apply, and this present ENM Guidance would no longer apply
4. Transformation during digestion	When evidence is provided convincingly demonstrating, by appropriate analytical methods that an ENM completely dissolves/degrades in the gastrointestinal tract, the hazard identification and hazard characterization can rely on data for the non-nanoform substance (if available) as long as the possibility of ENM absorption before the dissolution/degradation stage can be excluded. When evidence is provided convincingly demonstrating that no ENM absorption takes place a limited set of tests in general consisting of in vitro genotoxicity, in vivo local effects and/or other appropriate in vivo testing may be deemed as sufficient. The systemic toxicity profile of a dissolved ENM is likely to be similar to the soluble (ionic or molecular) form. If this is demonstrated, further testing on the ENM is not necessary. In cases where data on the non-nanoform are not available, testing of the non-nanoform is required according to the relevant EFSA Guidance for the intended use
5. Information on non-nanoform available	When information on a non-nanoform of the same substance is available and where some or all of the ENM persists in the food/feed matrix and in gastrointestinal fluids, a testing approach is recommended which is based on comparison of information on ADME, toxicity, and genotoxicity of the non-nanoform with, in first instance, ADME, repeated-dose 90-day oral toxicity study in rodents and genotoxicity information of the ENM. The purpose of comparing ADME and toxicity data from the two forms is to identify any major differences between the behavior of the non-nanoform and that of the ENM. If the differences observed indicate increased hazard, then more toxicity testing will be required on the ENM, beyond ADME, 90-day and genotoxicity tests. If the differences observed indicate less hazard then any request to waive further testing should be scientifically justified
6. No information on non-nanoform available	When information on a non-nanoform is not available and where some or all of the ENM persists in the food/feed matrix and in gastrointestinal fluids, the approach for toxicity tests on the ENM should follow the relevant EFSA guidance for the intended use with the modifications in the present guidance to take into account the nanoproperties. The ENM toxicity testing strategy provided for hazard identification and hazard characterization takes into account the nanoproperties

**Fig. 3:**
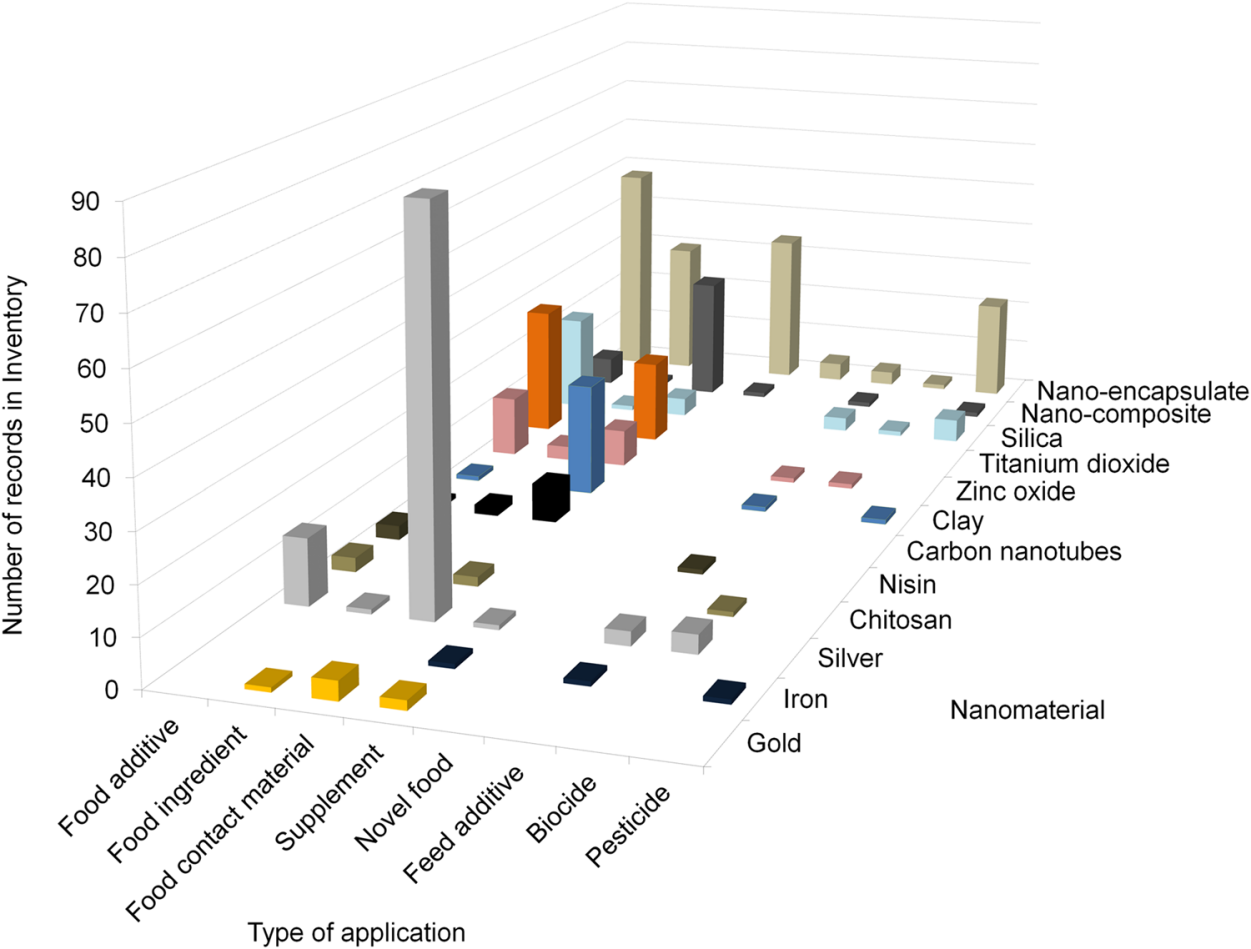
Information extracted from the Nano Inventory (Peters et al. 2014) shows the number of records of the most used nanomaterials in the most common types of applications. For reasons of clarity not all nanomaterials and applications are shown. “Silica” is the sum of synthetic amorphous silica and silicon dioxide

Despite current technical difficulties in food and feed risk assessment procedures EFSA evaluates the nanoscale-fraction solely on the basis of available information. An inventory of existing nanotechnology applications in the agricultural, feed and food sector revealed nano-encapsulates and Ag as the NMs most frequently used (RIKILT and JRC 2014). Food additives and food contact materials were asserted as the major fields of application (Fig. 3).

For risk assessment, EFSA demands a full report of physicochemical parameters and the respective analytical methods, the choice of which depends on the type of NM and the measurement environment. Applied methods should be demonstrated to be fit for purpose and suited to deliver reproducible results. Nevertheless, this represents a relatively new field in analytical chemistry. Therefore, it is without surprise that food labeling requirements, which have been imposed for transparency reasons (EC 2013), are hampered by the analytical challenges associated with the implementation of the EC recommendation for the definition of NMs.

It should be kept in mind that regulatory authorities will require reliable routine methods for any monitoring in compliance with the specification of the respective NM. Regarding safety studies with NMs used in food, members of the EFSA Nano Network note that unrealistically high dosing can lead to outcomes that may not be related to the inherent toxicity of the material but to the high amounts administered. Therefore, attention should be paid to the application of physiologically relevant test doses. Similarly as for materials used in cosmetics and food, the risk assessment of NMs according to the Novel Food Regulation (EC 2015) and the legislations for food contact materials (EC 2004) and food additives (EC 2008) involve case-by-case decisions as the current practice.

A specific challenge is the assessment of particle migration, e.g., pigments from nanocomposites which are increasingly used for food packaging but also in textiles. Similar to cleaning agents and detergents, textiles lack any nanospecific regulation, except for the applicability of REACH for their ingredients. However, the release of free nanoparticles from such everyday commodities that may come in contact with human skin or may be inhaled is considered a potential health concern because there is still uncertainty regarding hazardous local effects of particulate matter.

## Characterization of human exposure to nanomaterials

NMs are explicitly addressed in some sector-specific regulations such as for cosmetic products (EC 2009), and novel foods (EC 2015), which has triggered the need for analytical methods to detect and characterize them for regulatory purposes. It is expected that the slightly different definitions currently applicable for specific regulatory sectors will be harmonized with the EC recommendation on a definition of an NM. Nevertheless, it is necessary to implement the current definition and this has triggered research into the development of suitable methods. The NanoDefine project, for example, with its consortium of researchers, manufacturers, regulators and metrology institutes, aims to develop an integrated approach that combines different methods for particle size measurement and supplies practical guidance for their implementation. A method-driven material classification system is developed, differentiating mono- and multi-constituent substances as well as articles and consumer products. The project develops recommendations on sample preparation and measurement methods, depending on material type and purpose. Several methods are available to the project which can be grouped into counting, fractionation, ensemble and integral sizing methods that are applied in a tiered approach for screening, confirmation and eventually validation of the outcome (NanoDefine 2016). This means in practice, that, e.g., a chemical substance in nanoform may be characterized for registration under REACH by inductively coupled plasma mass spectrometry in single-particle mode (sp-ICP-MS) with a subsequent confirmation of results by electron microscopy. For cosmetics and food contact materials, additional considerations of method applicability to the complex matrixes of these products are required. The project aims to produce comprehensive guidelines to help the user select the most suitable and economic method(s) to decide, for a given regulatory purpose, whether a material is an NM according to the definition or not.

**Fig. 4:**
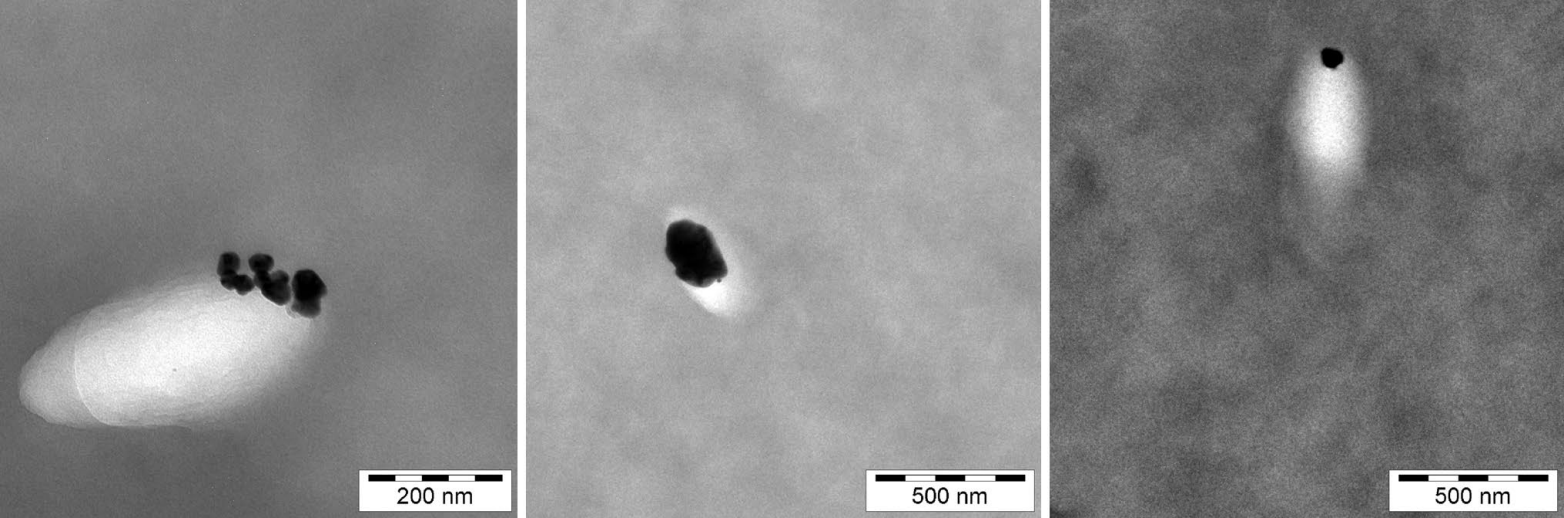
Migration potential of nanoparticles in food contact plastics: nano-silver in low-density polyethylene (LDPE)

For liquid and pasty matrices as well as in case of aerosols, human exposure can be assumed to be equal to the total number of nanoparticles applied with the product. The same is not true for polymer nanocomposites. Within composites, nanoparticles are embedded into a polymer matrix from which there need to be a release first for consumer exposure to occur. Therefore, the main question in case of nanocomposites for food contact materials is, if and under which circumstances nanoparticles can be emitted. Noonan et al. (2014) sort the possible release scenarios into four categories: (1) desorption from the surface for weak bonding; (2) diffusion in the polymer to the food contact surface; (3) dissolution into ions which are released into the food and (4) degradation of the matrix by abrasion, hydrolysis, swelling, etc. The migration potential of NMs from food contact plastics has been studied by numerous research groups yielding contradictory conclusions. Discrimination between particle release and migration of dissolved ions is crucial for proper interpretation of migration results (Noonan et al. 2014; Störmer et al. 2017). Furthermore, metal ions such as Ag^+^ can re-form nanoparticles at slightly reductive conditions, including during sample preparation (Störmer et al. 2017). Low-density polyethylene polymer (LDPE) has the highest diffusivity among usual food contact plastics (Bott et al. 2014a) and thus may be regarded as a worst-case matrix. To this example, for food contact plastics the ‘nano-additives’, Ag (Bott et al. 2014b), titanium nitride (Bott et al. 2014c) (Fig. 4), carbon black (Bott et al. 2014a), synthetic amorphous SiO_2_ (SAS) and laponite, a clay consisting of very small crystallites at the nanoscale (‘nanoclay’), were added at various concentrations. Migration testing for 10 days at 60 °C revealed a maximum release of titanium and Ag of 0.24 µg/kg and 1 µg/dm^2^, respectively, into the simulant acetic acid (3%). A lower migration was observed into ethanol (95%) and isooctane. In all cases, nanoparticles were undetectable in the simulants by inductively coupled plasma mass spectrometry (ICP-MS) and asymmetrical flow field-flow fractionation (AF4) (Störmer et al. 2017). In agreement with these findings, Bott et al. (2014c) have estimated by modeling that any migration would be below 1 × 10^−6^ mg/kg food using the conservative assumption of a nanoparticle size of less than 10 nm. Migration modeling indicates that nanoparticles larger than 3–4 nm in diameter cannot migrate at all from low-density polyethylene (LDPE). It has been concluded due to the high diffusion properties of LDPE, that migration of nanoparticles > 3 nm is unlikely from any other plastic food contact material following Fick’s law of diffusion (Maia et al. 2016). Based on the study of Bott et al. (2014c), EFSA has issued an opinion according to which consumer exposure to nanoparticles added to rigid polyvinyl chloride in concentrations of up to 10% (w/w) is expected to be very low and does not raise toxicological concerns (EFSA Scientific Committee 2014). However, consumer exposure to nanoparticles released from other polymer nanocomposites, e.g., functionalized textiles, is still insufficiently evaluated.

Due to the complex matrices encountered in toxicity and ecotoxicity testing, methods for sample preparation and particle characterization remain challenging and are still under development. Especially the differentiation between “persistent” and “non-persistent” particles, [e.g., by the EFSA exposure scenarios for ingestion (see Table 1)] or by the category “granular biodurable particle without known significant specific toxicity” (GBP) established by Roller and Pott (2006) for particle inhalation requires further data on the fate of the different materials. Only once reliable methods are available, these terms can be technically defined. Moreover, with the application of new techniques data are suggesting in vivo dissolution of particles that were previously considered biodurable, e.g., in the case for cerium dioxide (CeO_2_) (Graham et al. 2014; Moreno-Horn and Gebel 2014). On the other hand, reactive materials such as elemental aluminum whose oxides are water soluble (Shin et al. 2015) may acquire passivating shells (Padhye et al. 2016) that potentially could prevent a complete particle dissolution and lead to an unexpected long-term persistence of particles.

## Development of novel tools based on combination of multimodal spectroscopies to study cellular uptake processes

**Fig. 5:**
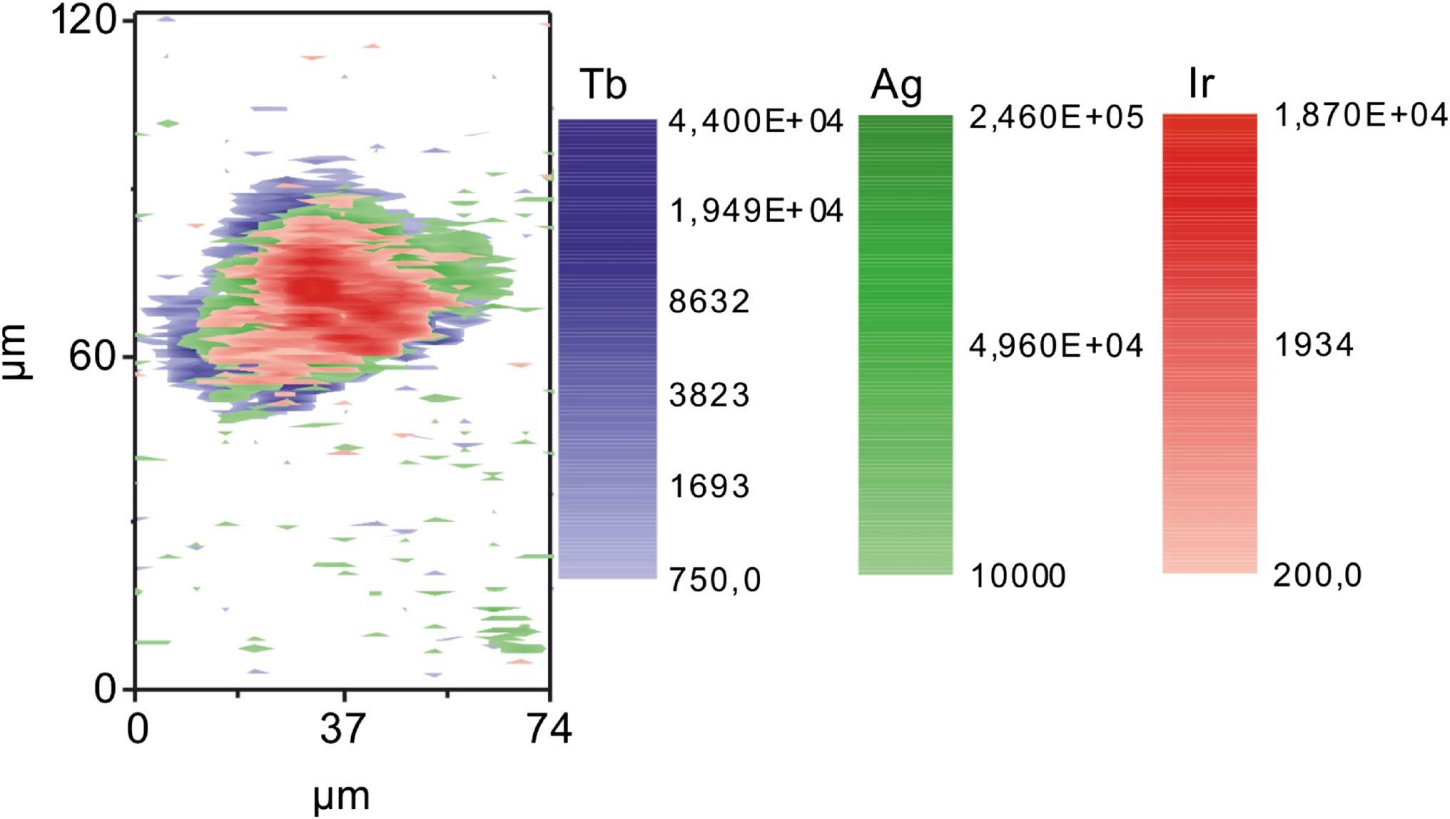
Analysis of silver nanoparticle distribution in individual cells by spatially resolved bio-imaging using laser ablation inductively coupled plasma mass spectrometry (LA-ICP-MS)

The quantification of NP uptake by different organs and their cells is essential for toxicological studies. Currently, for the intracellular quantification of NPs, analysis of a cell suspension or a pellet typically containing 10^6^ cells by ICP-MS is carried out (Hsiao et al. 2016). The method yields an average value and does not allow conclusions on the particle distribution among cells or within a single cell. For this reason, a method based on laser ablation, LA-ICP-MS, was developed in order to localize and quantify metallic NPs in single cells. Fibroblast and macrophage cells were incubated with Au or Ag NPs at different concentrations and grown under standard conditions. The NP distribution of individual cells was determined by spatially resolved bio-imaging using LA-ICP-MS (Fig. 5). Sub-cellular resolution was achieved by careful optimization of laser energy, ablation frequency and scan speed. Based on matrix-matched calibration, the number of NPs in individual cells was determined (Drescher et al. 2012, 2014).

In a more recent study, Au NPs have been used as probes for surface enhanced Raman scattering (SERS) to get molecular information from inside the cells. The gold NPs and their aggregates were quantified inside the cellular ultrastructure by LA-ICP-MS micro-mapping and evaluated regarding the SERS signals. In this way, both information about their localization at the micrometer scale and their molecular nano-environment, respectively, was obtained and can be related. Thus, the NP localization can be followed from endocytotic uptake, intracellular processing, to cell division. It was shown that the ability of the intracellular NPs and their accumulations and aggregates to support high SERS signals is neither directly related to NP amount nor to high local NP densities. The SERS data indicated that aggregate geometry and interparticle distances in the cell change in the course of endosomal maturation and play a critical role for the specific gold NP type in order to act as efficient SERS nanoprobe. This finding is supported by TEM images, showing only a minor portion of aggregates that present small interparticle spacing. The SERS spectra obtained after different chase times showed a changing composition and/or structure of the biomolecule corona of the gold NPs as a consequence of endosomal processing.

By adding a gold core to silica NPs (BrightSilica), silica-like NPs were generated that, unlike unmodified silica nanoparticles, provide three types of complementary information to investigate the silica nano-bio-interaction inside eukaryotic cells in situ. Firstly, organic molecules in proximity of and penetrating into the silica shell in live cells were monitored by SERS. The data show interaction of the hybrid silica particles with tyrosine, cysteine and phenylalanine side chains of adsorbed proteins. Composition of the biomolecular corona of BrightSilica NPs differed in fibroblast and macrophage cells. Secondly, quantification of the BrightSilica NPs using LA ICP-MS micro-mapping indicated a different interaction of silica nanoparticles compared to pure gold NPs under the same experimental conditions. Thirdly, the metal cores allowed for the investigation of particle distribution and interaction in the cellular ultrastructure by cryo-nanoscale X-ray tomography. In 3D reconstructions, the assumption was confirmed that BrightSilica NPs enter cells by an endocytotic mechanism. The results have implications for the development of multi-modal qualitative and quantitative characterization in comparative nanotoxicology and bio-nanotechnology (Buchner et al. 2016).

LA-ICP-MS cannot differentiate between particle adsorption to cell membranes or penetration into the cells; however, the technique is further capable to provide insight into the cell-to-cell variation of particle distributions (Hsiao et al. 2016).

Further development of the method toward an elemental microscope with a lateral resolution in the sub-micrometer range may allow for direct detection of NPs in cells and tissues. In perspective, the localization of NPs may be correlated with cellular compartments by using staining reagents or metal conjugated antibodies. In the first case protein and DNA distributions may be visualized (Herrmann et al. 2017) and in the second case dynamics of the cellular machinery may be determined (Mueller et al. 2017a).

LA-ICP-MS has already been applied to detect very small iron oxide NPs in tissues to answer the question if they can be applied for imaging of arterio-sclerotic plaques in magnetic resonance imaging experiments (Scharlach et al. 2016). Again, this method excels by simple calibration using just slurry suspensions of NPs to provide quantitative information. By doping the very small iron oxide NPs with rare earth elements even endogenous and exogenous iron can be differentiated. This may be used to provide information on local particle distribution in tissues and potential association with biomolecules if combined with the previously mentioned metal stains and/or metal-tagged antibodies.

However, the application of quantitative LA-ICP-MS in toxicokinetics is currently in a developmental stage. Recently, the technology “mass cytometry imaging” which is based on a laser ablation system coupled to inductively coupled plasma time-of-flight mass spectroscopy was launched (Mueller et al. 2017b). To summarize, the technology convinces by application of metal-tagged antibodies which are applied to thin-cuts of tissue samples so that up to presently 50 individual biomarkers can be detected simultaneously together with endogenous metals being present in different organs.

**Fig. 6:**
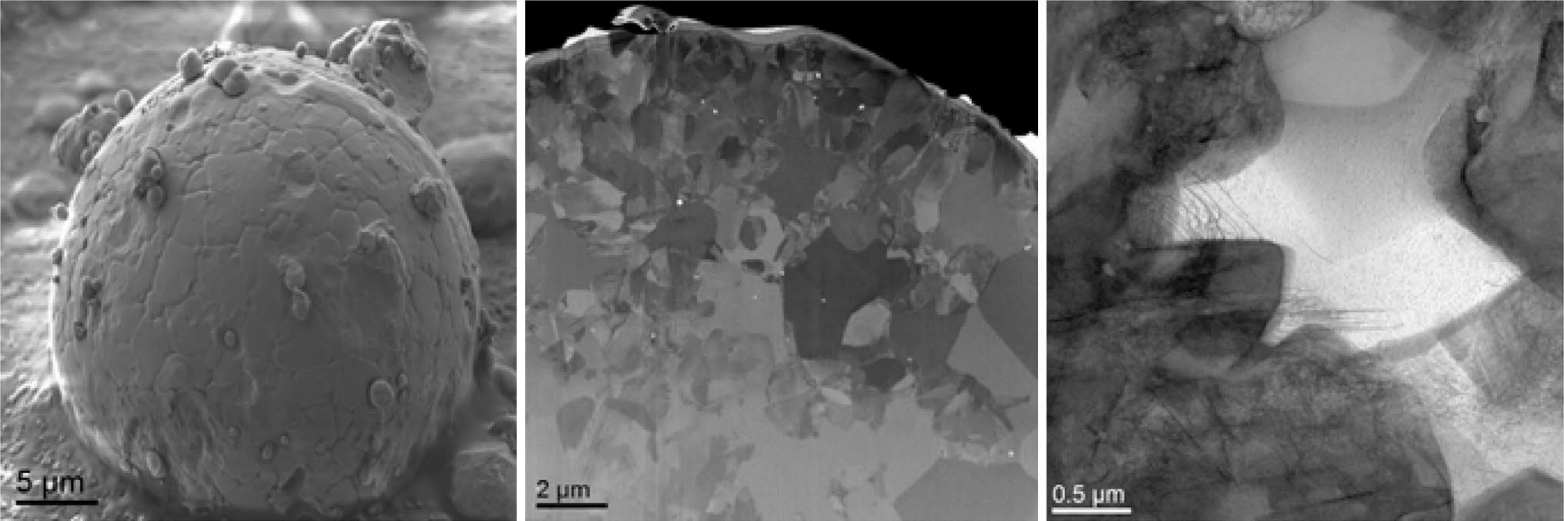
Investigation of surface structure (left), internal structure (center) and lattice defects (right) in combination of scanning electron microscopy (SEM), focused ion beam (FIB) and transmission electron microscopy (TEM)

Alternative imaging techniques to LA-ICP-MS on the other hand exhibit the potential to visualize particles on tissue and cellular level (Jungnickel et al. 2016; Laux et al. 2017a). A protocol for transmission electron microscopy (TEM) has been developed that allows to describe the mechanism of NP transport in a blood–brain barrier model and Caco-2 intestinal epithelial cells model (Ye et al. 2015). As it was recently shown by Graham et al. (2014) for CeO_2_, particle characteristics such as crystal structure, composition, and size may impact on toxicity. Recently developed new techniques such as focused ion beam (FIB) microscopy in combination with TEM or SEM allow the visualization of surface details and internal particle structures, respectively (Fig. 6) (Guehrs et al. 2017). These features could help to further elucidate in vivo transformation of internalized material. Furthermore, the benefit of coupling electron energy loss spectroscopy to TEM was recently proven for identification and morphological characterization of inhaled TiO_2_ NPs in the rat lung (Kapp et al. 2004).

## Toxicity and ecotoxicity testing of nanomaterials

So far, NM safety research is mainly focused on exposure via inhalation and ingestion. Indeed, the likelihood of NP uptake via the skin is considered smaller based on data obtained exemplarily with certain forms of TiO_2,_ even when the skin is damaged due to sunburn (SCCS 2014). However, dermal uptake following to chronic exposure or injuries cannot be excluded and requires further investigation, in particular when materials other than TiO_2_ are applied. The highest concern for human health is attributed to the inhalation of NMs (Borm et al. 2006).

Prior to any toxicological testing a complete and careful characterization of the NM is required. Doak et al. (2009) have suggested a three-phase approach for nanoparticle characterization in order to address this issue. Phase one covers the powder state and includes assessment of chemical composition, size, size distribution, surface area and morphology. Phase two covers characterization of the dispersions and includes tests on agglomeration or aggregation, as well as on the formation of reactive species. Phase three then includes the nano–bio interface, which needs to be assessed in a relevant biological medium.

In addition to a comprehensive characterization of the test materials and the employed media, an appropriate dosimetry is a crucial prerequisite for a consistent quantification of the material to which an animal or cellular system is exposed and thus the establishment of realistic exposure scenarios (Oberdörster 2012). The different outcomes of in vivo and in vitro toxicity testing achieved so far are considered to be in significant part due to a missing dose correlation between the systems (DeLoid et al. 2015; Demokritou et al. 2013). For the simulation of exposure to inhaled NMs in occupational and other scenarios, including animal experimentation, the multiple-path particle dosimetry model has been developed and is commonly used today (Anjilvel and Asgharian 1995; Cassee et al. 2002).

### Aerosolization techniques

Because of their high diffusivity, nanoparticles tend to agglomerate in air (Wong et al. 2009) and only a small percentage of respirable particles in aerosols exhibit a size of below 100 nm (Ma-Hock et al. 2007). Measurements at three different workplaces exposed to SiO_2_, carbon black and CaCO_3_ nanoparticles, respectively, revealed number and mass concentrations close to the background level (Tsai et al. 2011). A case study, addressing the nanoparticle release from nanoreinforced tires revealed an importance of parameters controlling release in the order: aging scenario, matrix properties, NM properties (Wohlleben et al. 2016b). In realistic scenarios, low levels of nanoparticles, but more so NM aggregates have been shown to become available in aerosols (Landsiedel et al. 2012). In addition to the main influence of concentration, primary particle size and the material-specific agglomeration status (Ma-Hock et al. 2007), the method applied for aerosolization impacts significantly the tendency of nanoparticles to agglomerate in air and is thus a key parameter for the biokinetics of nanoparticles. The generation of aerosols from powder dispersions by a brush dust feeder is considered more similar to environmental and workplace scenarios than the use of nebulization systems employing liquid vehicles, in particular as these may exert additional toxicological effects (Ma-Hock et al. 2007). Spark discharge, a technique widely used in material research and production (McKinney et al. 2009; Meuller et al. 2012) provides individual or slightly agglomerated nanoparticles that may be applied at very low concentrations. This enables progression of particles into the deeper lung where they may deposit and potentially might penetrate the air-blood barrier. Spark discharge has been used to demonstrate that iridium particles with a median diameter of 80 nm are translocated from lung to secondary organs to about an order of magnitude less in comparison to iridium particles with a median diameter of 15 nm (Kreyling et al. 2002). However, even though there are multiple applications in research and production, the relevance of such exposure scenarios for daily life scenarios remains questionable. While at one hand particle generation by spark discharge may reveal specific size-dependent differences in NM biokinetics, dry dispersion of the bulk material, e.g., by a brush generator, nozzles, or acoustical feeder systems (McKinney et al. 2009) represents a risk-related approach.

Respirability of the aerosol as a prerequisite for inhalation toxicity studies is determined by the particle size distribution which can be assessed by cascade impactors as well as by scanning mobility or aerodynamic particle sizers (Asbach et al. 2009; Ma-Hock et al. 2007). For inhalation studies with carbon nanotubes, measurement of tube length, curvature and diameter by electron microscopy are further necessary requirements due to the relationship between material structure and pulmonary response (Oberdörster et al. 2015). Recent data indicate that rigidity may represent a crucial factor for the carcinogenic potency of carbon nanotubes (Nagai et al. 2011). Test methods are not yet established and are subject of ongoing research (BAuA 2015).

### Testing for inhalation toxicity of nanoparticles

As shown for the example of coal dust which was detected in 10.4% of liver and 19.5% of spleen samples of coal mine workers (LeFevre et al. 1982), systemic availability of inhaled particles is not necessarily restricted to nanoscaled objects. Furthermore, no evidence for a relevant nanospecific toxicity has been provided so far (Moreno-Horn and Gebel 2014). However, engineered NMs consisting of different compounds may further possess altered toxicological properties like, e.g., nanosized cerium dioxide particles that did not cause lung inflammation after short-term inhalation when coated with amorphous silica (Demokritou et al. 2013; Gebel et al. 2014).

As an example for granular nanoparticles of low solubility, three particle types of the widely used TiO_2_ with different surface properties were compared regarding their effects and toxicokinetic fate in a 28-day inhalation study in rats, employing a dry dispersion technique (Eydner et al. 2012). Besides minimal inflammatory changes in the lungs, leucopenia, and a decrease in beta-glucuronidase, particle deposition in alveolar macrophages and, to a lesser extent, in type-I pneumocytes, was observed. A minimal translocation of particles into the bloodstream was described; the concentration of the substance was below the limit of detection in all other organs than lung. Similarly, nanoscaled zinc oxide and SAS were not detected in significant concentrations in other organs than lung and lung-associated lymph nodes following 90 days inhalation in rats. However, for both compounds dissolution is decreasing the lung burden in addition to the physiological clearance (Creutzenberg 2013, 2014).

In general, the size of inhaled agglomerates is considered a main determinant of the biokinetic fate of particulate materials (Eydner et al. 2012; Wiench et al. 2012). The application of particle agglomerates with a mass median aerodynamic diameter in the respirable range in most inhalation studies might explain why the effects of particulates are comparable, independent of their primary size (Gebel 2012). A factor of 2–2.5 referring to the dose metrics mass concentration was described between the two with regard to their carcinogenic potency (Gebel 2012). Positive results of chronic studies on tumor formation due to inhalation of carbon black and TiO_2_ provide sufficient evidence for inhalation carcinogenicity of both materials according to the International Agency for Research on Cancer (IARC 2010). However, because of the high doses that were applied it is questioned if substance specific conclusions can be drawn. So far, no regulatory classification of these substances has been accomplished. Any observed formation of lung tumors has been attributed to substance-independent, unspecific effects of overload, impaired lung clearance and subsequent inflammation (Baan 2007). This mechanism is hypothesized for GBP, a class of materials comprising compounds such as TiO_2_, carbon black or CeO_2_ (Roller and Pott 2006), regardless of their particle size (Gebel et al. 2014). The influence of surface functionalization on agglomeration of SiO_2_ and zirconium dioxide (ZrO_2_) was analyzed within the German project NanoGEM (2010) in different lung-related biofluids. Besides pure phospholipids, a commercially available extract from pig lungs (CuroSurf™) and purified native porcine surfactants were employed. Furthermore, lipid and protein interactions have been analyzed. Lipid binding was surprisingly low for pure phospholipids and rather seems to be mediated by proteins, with surfactant protein A being the most important one. A correlation of in situ characterization data to toxicological data obtained in short-term inhalation studies of the same nanoparticles (Landsiedel et al. 2014) revealed that differences in inhalation toxicity are linked to the nanoparticle core composition together with the surface area adsorbing lung surfactant, and not as much to the corona, neither lipid nor protein (Wohlleben et al. 2016a). However, protein and/or lipid binding may have a modulatory effect.

While many different potential effects such as inflammation, developmental toxicity, neurotoxicity and immunological reactions (Ema et al. 2017; Giannakou et al. 2016; Heusinkveld et al. 2016; Kermanizadeh et al. 2015; Zhang et al. 2015), have been unveiled in recent research, inhalation carcinogenicity of nanoparticles was flagged as a main question of nanotoxicology (Becker et al. 2011). A subchronic, 90-day inhalation study on toxicity and carcinogenicity aligned with a 90-day post-exposure period, investigated nano-CeO_2_ as a representative of the GBP group. An impaired lung clearance and a slight increase of genotoxicity and cell proliferation markers in comparison to the control were revealed for the highest dose group exposed to 3.0 mg/m^3^ CeO_2_. However, due to the small increase and the possibility of overload-related effects, the data on genotoxicity should be considered with care (Schwotzer et al. 2017). In order to evaluate the relevance of overload related effects, the requirement of a post-exposure period is currently considered for inclusion into the technical guidelines of the OECD working party on manufactured NMs for inhalation studies on nanoparticle toxicity (OECD 2015).

### Testing for inhalation toxicity of nanofibers

In deviation of the data for spherical particles, there is clear evidence for pulmonary and intraperitoneal carcinogenicity of biopersistent nanofibers such as certain MWCNTs (Donaldson et al. 2010; Porter et al. 2010; Rittinghausen et al. 2014), but also fibers from inorganic materials such as fluoro-edenite or silicon carbide (Grosse et al. 2014). Moreover, pulmonary fibrosis has been highlighted as an effect of MWCNT exposure (Sharma et al. 2016; Vietti et al. 2016). Intraperitoneal injection, described as the most sensitive method for carcinogenicity testing of fibers (Bernstein et al. 2001; Drummond et al. 2016) has revealed a relation between curvature and carcinogenic potency of MWCNTs. However, due to its sensitivity this method is useful for hazard identification and studying mechanisms of action, its applicability to risk assessment remains to be established. The induced mesotheliomas were characterized as similar to those revealed with asbestos (Rittinghausen et al. 2014). Frustrated phagocytosis (Dostert et al. 2008) and retention in the mesothelial stomata (Poland et al. 2008) as elements of the fiber paradigm are possible mechanisms of carcinogenicity for these materials. In case of MWCNTs a relation between shape and carcinogenic potential has been described: while thin, needle-like MWCNTs of 37–85 nm in diameter and lengths between 5.29 and 10 µm elicited a carcinogenic effect, thick or tangled MWCNTs with a diameter of 150 nm and a length of 4.88 or 4.34 μm, respectively, were less potent (Nagai et al. 2011; Rittinghausen et al. 2013). Similarly, as in the case of synthetic mineral fibers (Bernstein et al. 2001), biopersistence in the rat lung seems to be a suitable determinant for inhalation carcinogenicity of MWCNTs and other persistent nanofibers. There is the need to investigate in vitro and early-stage in vivo reactions as potential predictive markers for development of lung cancer or mesothelioma; the impact of physico-chemical properties and experimental factors should be considered (Kuempel et al. 2017). Some mineral fibers may dissolve in the bronchoalveolar lavage, dependent on the specific fiber properties (Nguea et al. 2008). An evaluation of man-made mineral fibers by the International Agency for Research on Cancer came to the conclusion that epidemiological studies only provide inadequate evidence for carcinogenicity in humans. Based on data from animal studies, sufficient evidence is seen for carcinogenicity of “Special-purpose” glass fibers and refractory ceramic fibers, while there is lower concern for insulation glass wool, rock (stone) wool and slag wool (Baan and Grosse 2004). Several attempts were taken in the past to increase the biosolubility of glass and stone wool compositions (Guldberg et al. 2000). However, so far man-made mineral fibers were mainly tested without the binder substances such as phenolic resin that are present in marketed products. Recent data achieved by abiotic methods indicate a strong influence of such compounds on fiber dissolution and suggest the testing of products as marketed in order to avoid human health risks (Wohlleben et al. 2017).

### Testing for oral toxicity

NMs may be ingested as food ingredients, novel foods, or compounds released from functionalized food contact materials. SAS is a well-known food additive (E551) (Napierska et al. 2010) and TiO_2_ a common whitening agent (E171); both comprise a fraction of particles in the nano-range (Peters et al. 2012; Weir et al. 2012). The biocidal properties of nano-Ag are used in kitchen equipment and water purification (Marambio-Jones and Hoek 2010). While for the rather insoluble materials SiO_2_, iron oxide and TiO_2_ no systemic toxicity and no or only negligible biodistribution were reported following oral administration to rats (Geraets et al. 2014; Yun et al. 2015), there are reports on a dose-dependent systemic distribution of ZnO and Ag nanoparticles, with liver, spleen, and lung as main targets (Choi et al. 2015; van der Zande et al. 2012; Yun et al. 2015). Exposure to Ag suspected to have genotoxic potency (Fewtrell et al. 2017) resulted in increased serum alkaline phosphatase and calcium levels as well as an enhanced concentration of the element in several tissues (Yun et al. 2015). Since also particles were detected following exposure to Ag^+^ ions, the systemic distribution of Ag was suggested to be due to the portion of soluble Ag^+^ salts (van der Zande et al. 2012). Possibly triggered by the expected higher potential of smaller nanoparticles to reach the nucleus via its pores of 8–10 nm in diameter (Magdolenova et al. 2014), there is the pending question whether there is a size-specific genotoxicity. For four particle types of commercially available SAS, no DNA damage was observed in seven tissues of rats orally exposed for a short period (Tarantini et al. 2015a). TEM images of human intestinal Caco-2 cells following exposure to two SiO_2_ nanoparticles of 15 and 55 nm size showed that both particle types were present in the cytoplasm but not in the nucleus. Chromosomal damage and release of the proinflammatory cytokine IL-8 was observed at the highest dose for 15 nm particles, but not for 55 nm particles, indicating the role of particle number and surface area in NM toxicity (Tarantini et al. 2015b). Data published on TiO_2_ nanoparticles are numerous and controversial (Zhang et al. 2015). Contradictory results are probably in part due to the materials photoactivity and the varying degree to which this was considered in the individual experiments. In vitro genotoxicity was reported by comet and micronucleus assays on modified HepG2 cells (Lichtenstein et al. 2015; Shukla et al. 2013). Several papers claimed that anatase crystalline structure nanoforms are more potent in inducing cytotoxic and genotoxic responses than rutile structures (Petkovic et al. 2011). Nevertheless, principles of the relationship between physico-chemical properties and toxicity have not been established so far.

Currently, interpretation of data on NM genotoxicity is hampered by often insufficient characterization of the NMs and the studies having been carried out under different experimental conditions. Several earlier in vitro genotoxicity results lack sufficient reproducibility. While, e.g., in the micronucleus assay an interference of reagents with cellular uptake of nanoparticles might reveal false negatives (Doak et al. 2009; Magdolenova et al. 2014), an interaction between nanoparticles and naked DNA following to cell lysis was described as a risk of artifacts during the comet assay (Stone et al. 2009). These experiences have led to improved protocols with better reproducibility, useful in a standard battery of test methods (Gonzalez and Kirsch-Volders 2016; Karlsson et al. 2015) and applicable to high-throughput screening (Collins et al. 2017). However, it remains unsolved whether genotoxic effects are direct or secondary to NM exposure, if this is not material-dependent in the first place (Evans et al. 2017).

In vivo genotoxicity must be investigated not only on systemic organs which may be exposed to low levels of nanoparticles depending on the bioavailability, but also on stomach and intestine which are the main organs in contact with food ingredients. Further research into agglomeration behavior and cellular uptake of nanoparticles (Tarantini et al. 2015a) as well as a standardization of tests are necessary in order to generate reliable information on NP genotoxicity. Apart from the as-produced material properties, different chemical environments of saliva, gastric juice, and chyle may cause dissolution or modification of particles and thus impact on barrier penetration and toxicity (Böhmert et al. 2014). The conditions of the gastrointestinal tract are considered by the EFSA exposure scenarios, according to which a material should be evaluated before it is used in agriculture, food and feed (EFSA 2011). Nano-Ag was shown to still occur in particulate form after digestion without major aggregation, indicating a potential for penetration of the intestinal barrier (Böhmert et al. 2014). The French/German project SolNanoTox intends to compare the effects of both, insoluble and soluble NPs by application of in vitro and in vivo models of intestine and liver. While the rutile forms of TiO_2_, which have been less investigated in oral settings so far, serve as an example of insoluble nanoparticles, aluminum is used as a soluble material.

### Ecotoxicity testing of nanomaterials

Similar to inhalation and oral toxicity assessment, agglomeration behavior and appropriate protocols for dispersion and substance delivery are also major points of discussion for the environmental testing of NMs. For dispersion, water without dispersant, stabilizer or dissolved organic matter is preferred (Laux et al. 2017b). As long as we do not know the most sensitive compartment, it is recommended to consider the three environmental compartments water, sediment and soil for the estimation of no observed effect concentrations (NOEC), unless exposure or ecotoxicity can be excluded. Due to NM properties such as agglomeration NMs may provoke the strongest effects at less than the highest dose. Therefore, limit tests are not recommended and several NM concentrations should always be tested. Some NMs such as TiO_2_ exhibit intrinsic photocatalytic activity or are (further) designed for this effect and show increased aquatic toxicity when relevant wavelengths are applied (Adams et al. 2006). However, even materials without such properties may be more harmful under illumination, as shown for nano-Ag on the survival of fish embryos (George et al. 2014). Accordingly, testing should include both conventional illumination and simulated sunlight. For hazard assessments, test conditions causing highest ecotoxicity should be selected. Environmental conditions such as aging, weathering or sewage treatment may cause modifications or altered bioavailability of NMs. Adsorption of matter from the environment can lead to corona formation with a potential change of ecotoxicological potency. For nano-Ag NM 300-K, a median effective concentration of 0.14 mg/L was observed at 48 h post-fertilization for particles in the effluent of a model sewage treatment plant in contrast to 1.09 mg/L for the pristine particles in mineral medium.

The exposure concentration for aquatic test organisms can also change as a result of sedimentation. This has to be considered when calculating effect concentrations. It must also be taken into account that organisms such as daphnids, though living in the water phase, can take up sedimented particles.

In soil and sediment tests the solid media may be spiked by applying soil or SiO_2_ sand as a carrier (dry spiking), or by using an aqueous dispersion of the NMs. A comparison of five spiking procedures using TiO_2_ and Ag NPs in standardized OECD tests resulted in stronger effects by wet spiking in comparison to dry spiking. Since there was an influence of stock suspension concentrations on the results observed, dry spiking was concluded as preferential method for application of solid TiO_2_ and Ag nanoparticles (Hund-Rinke et al. 2012). An essential topic in the ecotoxicological testing of NMs is aging and transformation over time. Amongst others, this may include processes such as photochemical transformation, dissolution, abrasion and biotransformation (Mitrano et al. 2015). Amorphous materials might be transformed in environmental media causing effects after prolonged incubation periods (Batley et al. 2013). The discussion on whether the incubation periods recommended in the test guidelines need to be modified for NM testing is ongoing. Based on laboratory data obtained with nano-Ag and nano-TiO_2_, firm proposals on the modifications of eight OECD test guidelines were established (Hund-Rinke et al. 2016), including testing of the green algae *Raphidocelis subcapitata* (OECD 2011), the sediment organism *Lumbriculus variegatus* (OECD 2007) and terrestrial invertebrates *Enchytraeus crypticus* and *Eisenia fetida* (OECD 2004a, b). A particular challenge is the assessment of ion-releasing NMs, for example when assessing their effect on the microbial nitrogen transformation in soil. In chemicals assessment the procedure described in OECD TG 216 (2000) is usually applied where Lucerne meal is used as complex nitrogen source. However, effects of ion-releasing NMs on nitrifying microorganisms are only detected if an inorganic nitrogen source is used instead (Hund-Rinke and Schlich 2014). Possible reasons are ions released from NMs sorb to organic nitrogen sources reducing their bioavailability, or that the oxidation sites of the NMs are blocked by the organic matrix. Therefore, transformation of NMs and their potential interaction with test media is an important aspect to be considered in order to avoid misinterpretation of potential effects.

## Risk communication and technological impact assessment

Nanotechnology is a key enabling technology of the early twenty-first century. However, while the forecasts between 2000 and 2010 of market volumes between 1 and 3 trillion USD (Thielmann 2015) were enthusiastic, expectations have calmed down meanwhile, as what is a usual trend for new technologies. Companies often focus on the best solution function- and cost-wise, somewhat restraining the launch of novel products. Worldwide, nanotechnology-based consumer products are mainly found in applications for health and fitness, home and garden and automotive, followed by food and beverage and coatings (Vance et al. 2015). Nano-Ag is one of the materials used most frequently and can be found in 25% of the nano-enabled products. Antimicrobial protection is representing its major purpose. It is assumed that the development of nanotechnology is following a double boom in specific publications and patents. Stagnation of scientific trends prior to the first patent boom is followed by an acceleration before the second patent boom. Usually, scientific activities are less fluctuating than patent activities, since companies react more rapidly when expected results are not realized. For a technology cycle, typically 15 years or more are expected between first and second boom. No substantial markets are existing before the second technological boom (Thielmann et al. 2009). Subsequent to the current phase of new orientation of consumers, enterprises and research, a broad diffusion of new product properties is expected within the next decades. Acceptance of nanotechnology is strongly related to the respective stakeholders. In some large companies concern has been raised against nanospecific labeling and information because of potential stigmatization and financial burden. In contrast, small and medium enterprises as, e.g., paint industries need more information on the NMs in use, while non-governmental institutions expect transparency. Finally, consumers might forget what nanotechnology is and may not differentiate to other products (Thielmann et al. 2009).

To further develop nanotechnology responsibly requires a broad continuous inclusion and integration of all stakeholders and their perspectives. The process should be moderated by neutral parties. Adopted measures of risk management should be taken, depending if free, bound or embedded particles are present. An assessment of the perception of nanotechnology of the public and the media was performed by the NanoView project (Epp 2015). While 41% of Europeans are positive about nanotechnology, 40% are still undecided (European Union of 27 member states in 2010). The awareness of nanotechnology is different at the country level. While it is 76% in Switzerland, 65% in Germany and 62% in the USA, it is only 46% on average in the 27 member states of the European Union. The willingness to buy nano-enabled products decreases when the expected intensity of human exposure increases. While surface coating and care products achieve the highest acceptance value of above 70%, acceptance decreases from textiles with 60% via cosmetics with 30% to a minimum of below 20% for food. The coverage of nano-enabled products in the German media was compared in a media analysis of the two periods from 2000 to 2007 and 2008 to 2012. The total number of media articles was 1696 in the first period and declined to 591 in the second. In contrast, the percentage of articles placed in the scientific sections of newspapers and news magazines increased from 58.5% between 2000 and 2007 to 66.5% between 2008 and 2012 (Epp 2015). Most articles highlight at least one benefit in relation to nanotechnology which corresponds to the rather positive overall risk–benefit perception in the German public. However, nanotechnology is not a frequently raised issue in the German public, but has become a subject of a highly specialized scientific discourse instead. An ongoing social discourse among various stakeholders about risks and benefits of nanotechnology together with a scientific risk assessment of NMs may help to further raise the awareness for nanotechnology and its applications in a responsible manner.

## Conclusions

The major role of high-volume NMs such as carbon black and SiO_2_ in industrial production is accompanied by regulatory and standardization measures. These require appropriate analytical capability for material characterization, which is still in development for some applications. Results of toxicological inhalation studies indicate a gradual increase of NM toxicity rather than completely new nano-specific effects. Such an effect should also not be expected due to the rather arbitrary (and differing) nature of the definition of the term nanomaterial. However, even though the nanodimension as such may not present a toxicological hazard, the large amount of new materials, in particular hybrid materials consisting of different compounds require further attention. Given the multitude of NMs in industrial processes and daily life, a further development of analytical techniques seems indispensable for (1) an accurate quantitative characterization of exposure scenarios; (2) the further elaboration of potential adverse effects on humans, and (3) the consideration of nanosized particulate matter with regard to its environmental fate and ecotoxicity. With regard to the first point, it might be worth taking the procedures successfully developed for testing of food and food contact materials as an example for the development of appropriate techniques that allow a characterization of human exposure by other daily life products such as cosmetics and textiles. For point two, the identification of adverse effects of nanosized particles, a further integration of biokinetic studies in toxicological testing schemes may help to reveal specific capabilities such as membrane penetration and intracellular accumulation. In consideration of the environmental fate and a potential impact of NM on ecotoxicity (3), it seems crucial to identify the alterations of an NM during its different life cycle stages as these may change the toxicological properties of a material. Adoption of analytical techniques such as LA-ICP-MS to biological matrices is promising. However, it is time-consuming and requires a stronger attention by projects on nanosafety. Independent of the question whether NMs exhibit a specific toxicity, a thorough characterization of toxicological test systems will help to improve data quality and understanding of toxicokinetics in general. This is a crucial prerequisite for reliable risk assessments and a broad common acceptance of novel technologies in the public.

## Abbreviations

AF4: Asymmetrical flow field-flow fractionation
EC: European Commission
ECHA: European Chemicals Agency
EFSA: European Food Safety Agency
ENM: Engineered nanomaterial
FIB: Focused ion beam microscopy
GBP: Granular biodurable particle without known significant specific toxicity
ICP-MS: Inductively coupled plasma mass spectrometry
ISO: International Organization for Standardization
LA-ICP-MS: Laser ablation inductively coupled plasma mass spectrometry
LDPE: Low-density polyethylene
NM: Nanomaterial
REACH: Registration:evaluation:authorization and restriction of chemicals
sp-ICP-MS: Inductively coupled plasma mass spectrometry in single-particle mode
SAS: Synthetic amorphous SiO_2_
SEM: Scanning electron microscopy
SERS: Surface enhanced Raman scattering
TEM: Transmission electron microscopy
UV: Ultraviolet radiation
